# Photogating Regimes in Graphene: Memory-Bearing and Reset-Free Operation

**DOI:** 10.3390/nano15211667

**Published:** 2025-11-02

**Authors:** Afshan Khaliq, Hongsheng Xu, Akeel Qadir, Ayesha Salman, Sichao Du, Munir Ali, Shihua Huang

**Affiliations:** 1 Department of Physics, Zhejiang Normal University, Jinhua 321004, China; 2 Department of Optoelectronics, Zhejiang Institute of Optoelectronics & Zhejiang Institute for Advanced Light Source, Jinhua 321004, China; 3 Industry-Education-Research Institute of Advanced Materials and Technology for Integrated Circuits, Anhui University, Hefei 230601, China; 4 School of Information Engineering, Xi’an Eurasia University, Xi’an 710065, China; 5 School of Electronics and Computer Sciences, Air University, Islamabad 44230, Pakistan; 6 College of Information Science and Electronic Engineering, Zhejiang University, Hangzhou 310027, China

**Keywords:** displacement current, deep-depletion, graphene channel current, graphene/SiO_2_/n-Si heterostructures, retention

## Abstract

We demonstrate photogating in a graphene/Si–SiO_2_ stack, where vertical motion of photogenerated charge is converted into a corresponding change in graphene channel conductance in real time. Under pulsed illumination, holes accumulate at the Si/SiO_2_ interface, creating a surface photovoltage that shifts the flat-band condition and electrostatically suppresses graphene conductance. A dual-readout scheme—simultaneously tracking interfacial charging dynamics and the graphene channel—cleanly separates optical charge injection (cause) from electronic transduction (effect). This separation allows for the direct extraction of practical figures of merit without conventional transfer sweeps, including flat-band shift per pulse, retention time constants, and trap occupancy. Interface kinetics then define two operating regimes: a fast, resettable detector when traps are sparse or rapid, and a trap-assisted analog-memory state when slow traps retain charge between pulses. The mechanism is complementary metal-oxide–semiconductor compatible (CMOS-compatible) and needs no cryogenics or exotic materials. Together, these results outline a compact route to engineer integrating photodetectors, pixel-level memory for adaptive imaging, and neuromorphic optoelectronic elements that couple sensing with in situ computation.

## 1. Introduction

Photogating [1,2,3,4,5,6,7], where optically generated charge stored in a remote region electrostatically modulates a conducting channel, has become a versatile route to high internal gain, low-power sensing, and history-dependent signal processing in emerging optoelectronics. Graphene [8,9,10,11,12,13,14] is a natural transducer for such effects: its semi-metallic density of states, high mobility, and atomically thin form factor make its conductance exquisitely sensitive to small gate perturbations, while its broadband transparency allows it to be paired with an underlying absorber rather than relying on direct absorption in the channel [15,16,17,18,19]. On silicon platforms, the native Si/SiO_2_ interface provides a well-characterized environment for surface-photovoltage, depletion and deep-depletion dynamics, and trap-mediated charge retention. These ingredients can both amplify weak optical stimuli and imprint short-term memory, with direct relevance to integrating photodetectors and neuromorphic elements [20,21,22,23,24,25,26,27].

Here, we implement a dual-readout graphene/SiO_2_/n-Si platform that measures, on the same device and in real time, the lateral graphene channel current $\left(I_{Gr}\right)$ [15,16] and the vertical displacement current ${(I}_{Dis})$ [28,29,30,31,32,33,34] while the silicon substrate is driven by a positive triangular ramp. By restricting operation to depletion/deep-depletion (i.e., avoiding negative bias and majority-carrier accumulation), we isolate minority-carrier integration in Si as the origin of photogating: under 915 nm illumination, photogenerated holes drift to and integrate at the Si/SiO_2_ boundary while electrons are swept into the bulk. The resulting interfacial charge shifts the MOS flat-band voltage ($V_{FB}$) and capacitively gates graphene across the 100 nm oxide, reducing its conductance at a constant bias voltage applied between graphene’s source and drain terminals ($V_{DS}=1\;V$). Recording $I_{Dis}$ and $I_{Gr}$ simultaneously thus provides both the time derivative of the interfacial charge (through $I_{Dis}$) and the state of that charge (through $I_{Gr}$), allowing us to validate the mechanism without heavy modeling.

A second ingredient is a pulse-within-ramp protocol that embeds multiple optical bursts within each gate sweep. This makes the photogate’s build-up directly visible as a staircase in $I_{Gr}$ synchronized with bursts in $I_{Dis}$, enabling in situ extraction of the light-induced flat-band shift $\Delta V_{FB}$ per pulse and per ramp from either the integrated displacement charge or the discrete channel-current steps—without recourse to separate DC transfer measurements. Applying the same metrology across nominally identical devices reveals a clear dichotomy governed by interface kinetics: one device exhibits trap-assisted retention (cycle-to-cycle $I_{Gr}$ baseline drift with attenuation of $I_{Dis}$ peaks), while a second device with a larger ramp range resets to the same baseline every cycle with stationary $I_{Dis}$ transients, indicative of a cleaner/faster Si/SiO_2_ interface. Together, these observations delineate a tunable continuum from instantaneous photogating to analog, photo-capacitive memory, controlled primarily by interfacial trap density and detrapping times.

Beyond establishing causality, the dual-readout approach yields compact, operation-level metrics. From voltage-domain sweeps we link slope and offset changes to interfacial charge, while time-domain traces quantify retention and reset dynamics. The methodology is simple—triangular ramping and modest optical power—and CMOS-compatible, making it attractive both as a diagnostic of interface quality and as a design tool for target behaviors. In the remainder of this article, we describe device fabrication and the physical mechanism, present time- and voltage-domain measurements (including pulse-within-ramp experiments) on two devices spanning retention and reset regimes, and discuss how ${\Delta V}_{FB}$ map onto interface engineering knobs and application spaces in integrating photodetection and neuromorphic optoelectronics.

## 2. Device Fabrication

An n-type Si/SiO_2_ (500 µm/100 nm) semiconductor substrate with a resistivity ranging from 1 to 10 Ω·cm, corresponding to a dopant concentration of approximately 4.5 × 10^14^−4.94 × 10^15^ cm^−3^, was utilized. The SiO_2_ dielectric layer was patterned through standard photolithography [35], followed by sequential thermal evaporation to deposit Cr/Au bilayer electrodes, with respective thicknesses of 10 nm and 80 nm.

Graphene synthesized via chemical vapor deposition (CVD) on a copper foil was spin-coated with a polymethylmethacrylate (PMMA) support layer. The underlying copper substrate was subsequently etched away in a CuSO_4_ + HCl + H_2_O solution for six hours. The resulting PMMA/graphene films were then rinsed in deionized water for two hours [36] and transferred onto the silicon wafer to cover the designated channel region and the Cr/Au contacts. PMMA removal was achieved using acetone, followed by cleaning with isopropyl alcohol (IPA). Graphene was subsequently patterned into 500 µm × 500 µm square shape using photolithography and oxygen plasma etching. The residual photoresist was removed with acetone and IPA to complete the device fabrication.

Afterwards, gold wire bonding was employed to connect the top electrodes for transient electrical and optical characterization. A customized measurement setup comprising a dual-channel signal generator, a power amplifier, a 915 nm pulsed laser, and an oscilloscope was used to record the $I_{Dis}$ and $I_{Gr}$ currents. To ensure consistency, the same set of measurements was also acquired using the Keithley 4200 semiconductor characterization system. During all optical measurements, the graphene/SiO_2_/n-Si heterostructure was illuminated vertically from the top surface.

## 3. Results and Discussion

### 3.1. Device Layout, Measurement Scheme, and Operating Band Diagram

**Figure 1:**
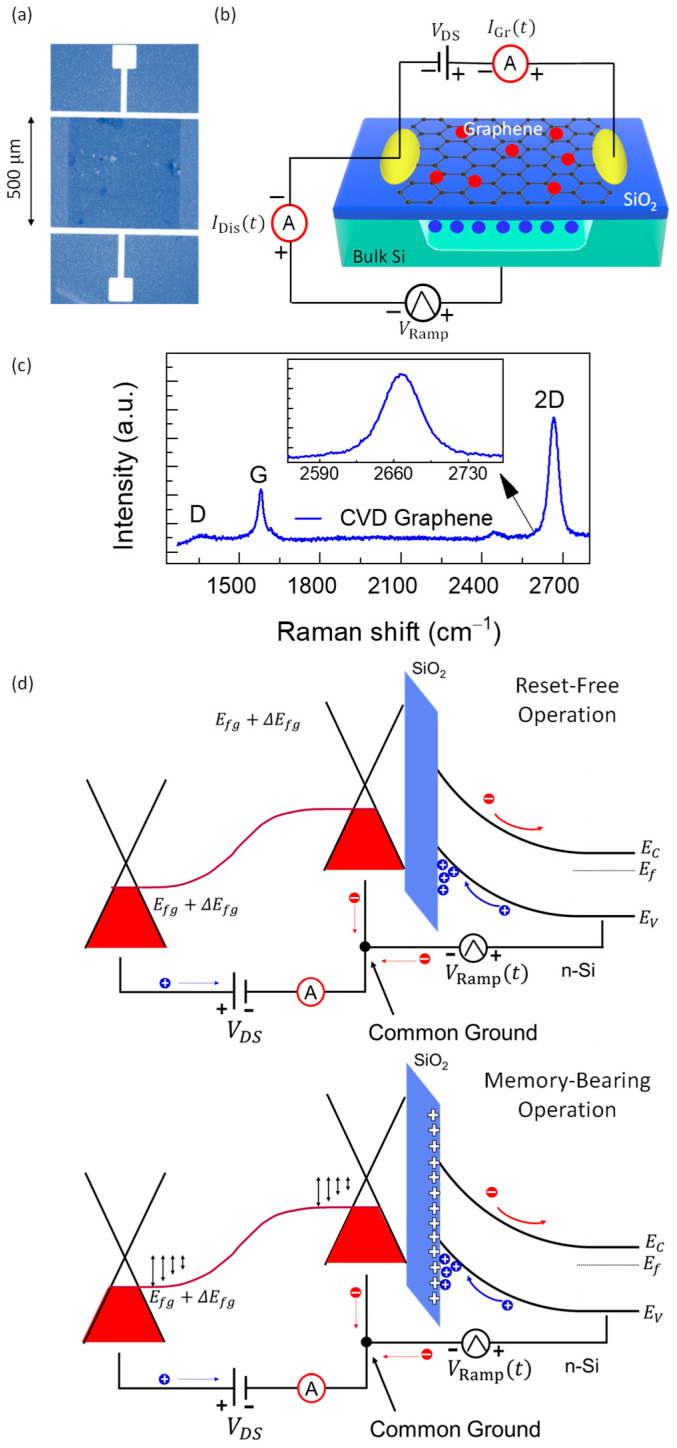
Device layout, measurement scheme, and operating band diagram. (**a**) Optical top view of the graphene/SiO_2_/n-Si device; graphene pad 500 µm × 500 µm. (**b**) Dual-readout: a positive triangular $V_{Ramp}$ (0–40 V or 0–93 V at $f_{r}=$1 kHz) is applied to n-Si relative to the grounded graphene source; graphene is biased with $V_{DS}=1\;V$ (drain + 1 V, source 0 V). The displacement current $I_{Dis}$ is measured at the Si terminal and the graphene channel current $I_{Gr}$ is measured laterally while illumination is at 915 nm. (**c**) Raman spectra to ensure the implementation of tunable monolayer CVD graphene. (**d**) Top: Band diagram under positive bias: depletion/deep depletion in n-Si; photogenerated holes accumulate at Si/SiO_2_, creating a positive interfacial charge $Q_{it}\;$ that shifts $V_{FB}$ and photogates graphene. Clean Si/SiO_2_ interface is prerequisite for reset-free operation of the graphene/SiO_2_/n-Si heterostructure. Bottom: The degree of charge retention due to slow traps at the Si/SiO_2_ interface ensures memory-bearing operation.

Figure 1 introduces the device, measurement scheme, and operating physics used throughout. Herein, Figure 1a shows the fabricated graphene/SiO_2_/n-Si heterostructure: a 500 µm × 500 µm graphene square which was contacted on opposite sides to form the lateral channel. Then, Figure 1b depicts the dual-readout configuration: a positive triangular $V_{Ramp}$ (0–40 V or 0–93 V at $f_{r}$ = 1 kHz) is applied to the n-Si photogate with respect to the grounded graphene source, while the graphene channel is read out at a fixed V_DS_ = 1 V (drain at +1 V, source at 0 V). The triangular back-gate ramp signals provide clear displacement-current signature ${(I}_{Dis\;}\propto dV/dt)$ while remaining slow enough to resolve interface-trap kinetics. The waveform avoids interfacial accumulation, preventing trap neutralization and enabling the observed retention. Illumination at 915 nm generates carriers predominantly in Si (minimal graphene absorption), supporting vertical minority-carrier integration with negligible heating. A lateral bias $V_{DS}=1\;V$ reads out the graphene channel in the linear regime with minimal Joule heating and contact artifacts.

Two currents are recorded simultaneously—vertical $I_{Dis}$ at the Si terminal (charging/discharging and photo-injection) and lateral $I_{Gr}$ through graphene (electrostatic transduction of stored interfacial charge). We measured Raman spectra [37,38,39,40,41,42] to probe the interface quality as presented in Figure 1c. The Raman spectra show tunable high-quality monolayer CVD graphene with large $I_{2D}/I_{G}\;>\;2$ and low $I_{D}/I_{G}<1$ peak ratios. Figure 1d gives the band-diagram view under positive gate bias: upward band bending in n-Si establishes depletion/deep depletion; 915 nm illumination generates carriers in Si, with holes integrating at the Si/SiO_2_ interface (depicted traps) and electrons swept into the bulk. The resulting positive interfacial charge $Q_{it}$ shifts the flat-band voltage and capacitively gates graphene, moving its Fermi level ($\Delta E_{fg}$) toward the Dirac point and thereby reducing $I_{Gr}$. Depending on interface kinetics, $Q_{it}$ either resets each cycle (memory-free device; Figure 1d: top) or partially persists (trap-assisted retention; Figure 1d: bottom), which explains the two operation regimes presented in this manuscript. Moreover, the observed retention is best explained by the net positive charge left near the Si/SiO_2_ interface or the effective emptying of donor-like interface states, which leaves them positively charged. Under employed positive ramp on n-Si, photogenerated holes drift to the Si/SiO_2_ boundary and a fraction is stored, producing a persistent positive photogate across the oxide. That positive interfacial charge constructs $\Delta E_{fg}$ and reduces $I_{Gr}$, and because part of it remains after the ramp, the conductance stays suppressed finally observed through retention.

### 3.2. Performance Parameters

A positive $V_{Ramp}$ on n-Si drives the MOS stack through depletion into deep depletion when the sweep outruns intrinsic minority-carrier supply. Under illumination, electron–hole pairs are generated, primarily in Si. The vertical field separates them: holes drift toward the Si/SiO_2_ interface and electrons into the Si bulk. The interfacial hole sheet charge $Q_{it}(t)$ builds during the positive sweep and partially decays on the return. The measured vertical current can be written as follows:

$$I_{Dis}\left(t\right)=C_{eff}(V)\frac{dV_{Ramp}}{dt}+\frac{dQ_{it}}{dt}$$

where $C_{eff}(V)$ is the voltage-dependent MOS capacitance. Sharp spikes in $I_{Dis}$ occur at ramp edges (rapid charging/discharging) and, when the light is pulsed. Integrating $I_{Dis}$ over time yields the transferred charge, $Q_{Ramp}=\int I_{Dis}\;dt,$ from which the photo-induced interfacial charge can be isolated after subtracting the quasi-capacitive background. Charge stored at the Si/SiO_2_ boundary shifts the MOS flat-band voltage, producing an effective back-gate perturbation on graphene ${\Delta V}_{FB}=\frac{Q_{it}}{C_{ox}}$, $C_{ox}=\frac{\varepsilon_{ox}}{t_{ox}}$.

This electrostatic shift modulates the graphene conductance, ${\Delta I}_{Gr}\approx g_{m\;}{\Delta V}_{FB}$, with $g_{m\;}$ the local transconductance of the graphene channel. In practice, $I_{Gr}$ decreases with increasing $V_{Ramp}$ because the positive photogate drives graphene toward charge neutrality.

If interface traps are present (density $D_{it}$) and/or have slow detrapping kinetics (time constant $\tau_{t}$ comparable to or longer than the 1 ms period), a fraction of $Q_{it}$ persists after the sweep, yielding: (i) a baseline offset in $I_{Gr}$ at the start of the next cycle and (ii) a cycle-to-cycle reduction in $I_{Dis}$ peak amplitude. Conversely, with a cleaner/faster interface (low $D_{it}$, $\tau_{t}\ll 1$ ms, the interfacial charge annihilates each cycle: $I_{Gr}$ resets to the dark baseline and $I_{Dis}$ peaks repeat perceived as instantaneous photogating without memory. The cumulative flat-band drift can be quantified by

$${\Delta V}_{FB}^{\left(Ramp\right)}=\frac{1}{C_{ox}}\int \left(I_{Dis}-C_{eff}\frac{dV_{Ramp}}{dt}\right)\;dt,$$

and its evolution with cycle index n is often captured by a stretched-exponential ${\Delta V}_{FB}\left(n\right)={\Delta V}_{FB}^{\infty }[1-e^{-{\left(n/n_{o}\right)}^{\beta }}]$ (trap-limited photo-integration). Furthermore, the millisecond-scale, polarity-dependent response that tracks the MOS field; the strong correlation between $I_{Gr}$ steps and $I_{Dis}$ spikes; the low optical power; and the sub-percent absorption in graphene at 915 nm collectively rule out the contributions from hot-carrier/bolometric or photothermoelectric as primary mechanisms.

### 3.3. Memory-Bearing Operation: Device A

**Figure 2:**
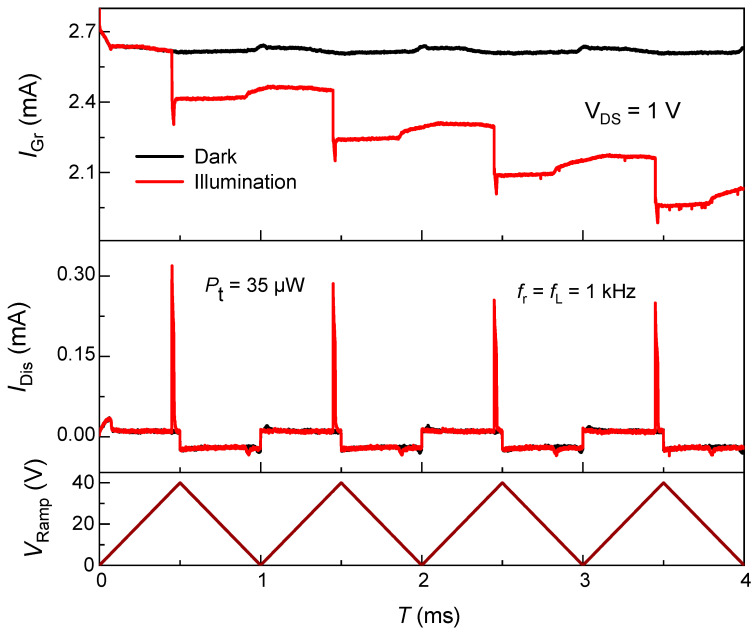
Deep-depletion photogating behavior in a graphene/SiO_2_/n-Si heterostructure. Top: Graphene channel current ($I_{Gr}$) at $V_{DS}=1\;V$ under dark (black) and 915 nm illumination (red) while a triangular ramp (0–40 V,$\;f_{r}=1\;kHz$) is applied to the n-Si substrate. Middle: Displacement current ($I_{Dis}$) collected perpendicular to the Si/SiO_2_ interface with graphene serving as the return electrode. Bottom: Applied ramp voltage ($V_{Ramp}$).

Figure 2 presents the time-resolved current response of a graphene/SiO_2_/n-Si heterostructure operated in deep depletion using a single-laser-pulse-per-ramp protocol. In each ramp cycle, an optical pulse generates electron–hole pairs predominantly in silicon. Under the positive gate ramp, the photo generated holes are driven toward the Si/SiO_2_ interface shifting the flat-band voltage ${\Delta V}_{FB}=Q_{it}/C_{ox}$ and capacitively gates graphene, moving its Fermi level toward the Dirac point and reducing its conductance. The result is a discrete, pulse-synchronized step decrease in $I_{Gr}$ (top panel, red). In the dark (black trace), $I_{Gr}$ remains essentially constant at ~2.7 mA throughout the ramp cycles, confirming negligible thermal generation and demonstrating the intrinsic stability of the graphene channel at $V_{DS}=1\;V$. Under illumination, $I_{Gr}$ exhibits periodic reductions that synchronize with each voltage ramp, evidencing a strong capacitive coupling between the integrated photocharge and the graphene channel. The distinct, repeatable transitions highlight the device’s fast and stable response to repetitive optical cycling.

The middle panel shows the corresponding $I_{Dis}$, which displays sharp, symmetric spikes correlated with the rising and falling edges of $V_{Ramp}$. These spikes mark the rapid charging and discharging events of the MOS capacitor during each ramp cycle. The gradual reduction in $I_{Dis}$ peak amplitude over successive cycles signifies trap filling and interfacial charge retention, which lower the effective capacitance and partially inhibit complete discharge. The concurrent non-recovery of $I_{Gr}$ to its dark baseline after each ramp cycle further substantiates persistent interfacial charging within both the Si/SiO_2_ and graphene/SiO_2_ interfaces. Since no negative bias is applied to induce accumulation, these trapped photo-generated holes lack an efficient recombination pathway, resulting in a residual positive gating field that sustains the conductance suppression even as the ramp returns to zero. The incomplete reset of $I_{Gr}$ and amplitude roll-off of $I_{Dis}$ reveal slow detrapping kinetics. Such behavior parallels photo-capacitive memory or short-term plasticity effects in neuromorphic phototransduction, indicating potential for integrating-type photodetectors and optoelectronic synapses where transient charge retention encodes temporal information.

Using the single-pulse charges $Q_{pulse}=\{$3.3, 3.1, 2.9, 2.7} nC and the device oxide capacitance $C_{ox}\approx 86\;pF$, the flat-band shift per pulse is ${\Delta V}_{FB,pulse}=Q_{pulse}/C_{ox}=$ 38 V, 36 V, 34 V, and 31 V for cycles 1–4, respectively. Treating the drop in injected charge between identical cycles as the minimum charge persisting to the next cycle gives $Q_{ret}^{\left(1\to 2\right)}=0.18\;nC,\;Q_{ret}^{\left(2\to 3\right)}=0.18\;nC,\;$ and $Q_{ret}^{\left(3\to 4\right)}=0.21\;nC$, corresponding to retained flat-band shifts of 2.1 V, 2 V, and 2.4 V. The associated retention fractions are 5.6%, 5.7%, and 7.2%, with charge ratios $R_{n}=\frac{Q_{n+1}}{Q_{n}}$ of 0.94, 0.94, and 0.93. Cumulatively over cycles 1→4, the retained charge is 0.57 nC, equivalent to a net retained ${\Delta V}_{FB}$ of 6.6 V, and a simple “memory index” $M=1-\;\frac{Q_{4}}{Q_{1}}$ equals 17.4%. The retained charge converts to occupied trap density as $N_{it}^{\left(1\to 2\right)}=4.6\times 10^{11}\;{cm}^{-2},\;N_{it}^{\left(2\to 3\right)}=4.4\times 10^{11}\;{cm}^{-2},\;$ and $N_{it}^{\left(3\to 4\right)}=5.1\times 10^{11}\;{cm}^{-2}$, with a cumulative occupied trap density of $1.4\times 10^{12}\;{cm}^{-2}$ over the four plotted cycles. These values represent conservative (lower-bound) retentions; any detrapping within a cycle would make the true persistent charge slightly larger.

**Figure 3:**
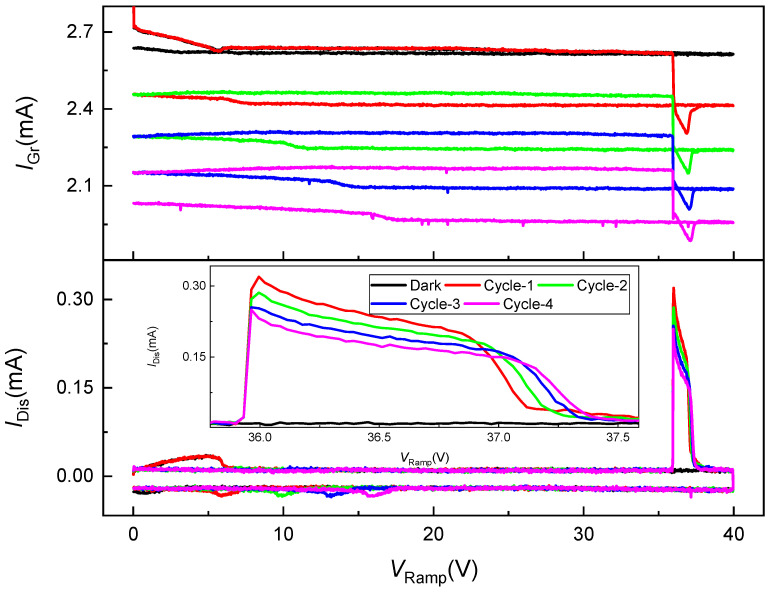
Voltage-dependent evolution of graphene and displacement currents under successive ramping cycles. Top: Graphene channel current ($I_{Gr}$) vs. applied ramp voltage ($V_{Ramp}$) at $V_{DS}=1\;V\;$ for consecutive ramp cycles under dark (black) and illuminated (colored) conditions. Bottom: Corresponding displacement current ($I_{Dis}$) traces collected through the Si/SiO_2_ stack, with the inset magnifying the 35–38 V region. All measurements were performed under 915 nm pulsed illumination ($P_{t}=35\;µW$) with a 0–40 V triangular ramp applied to the n-Si substrate at 1 kHz. Successive cycles (red → magenta) show progressive reduction in both $I_{Dis}$ peak amplitude and $I_{Gr}$ baseline, signifying interfacial trap filling and persistent photo-induced gating in the deep-depletion regime.

Figure 3 shows the dependence of both $I_{Gr}$ and $I_{Dis}$ on $V_{Ramp}$ (0–40 V) for several consecutive ramping cycles. The employed device, and illumination conditions are identical to those described for Figure 2. In the top panel, the dark current (black) remains nearly constant around 2.7 mA across the full voltage sweep, reaffirming the negligible influence of thermal generation. Under illumination, the graphene current decreases monotonically with increasing $V_{Ramp}$, forming distinct, nearly parallel traces for successive cycles (red → green → blue → magenta). The progressive downward shift of the $I_{Gr}-V_{Ramp}$ curves indicates accumulative photogating, arising from the gradual build-up of integrated hole charge at the Si/SiO_2_ interface. Because the biasing sequence avoids any negative voltages, no majority-carrier (electron) accumulation occurs to neutralize these holes; thus, the interfacial charge partially persists between cycles, shifting the graphene’s effective gate potential toward more positive values.

Each curve’s shape shows a gentle, nearly linear decrease in $I_{Gr}$ with increasing bias until abrupt transients appear near the ramp’s termination (~38−40 V), corresponding to the rapid collapse of the deep-depletion field when $I_{Dis}$ spike occurs. The absence of complete return to the initial dark baseline even at low $V_{Ramp}$ underscores trap-assisted retention and slow recombination kinetics at both Si/SiO_2_ and graphene/SiO_2_ interfaces.

The bottom panel and its inset depict the $I_{Dis}$ transients measured concurrently through the vertical path. As $V_{Ramp}$ increases, a pronounced charging peak emerges near 35–38 V, followed by a sharp discharge upon ramp reversal. The inset magnifies this regime, revealing that the first cycle (red) produces the largest $I_{Dis}$ peak (~320 µA), whereas subsequent cycles exhibit systematically reduced amplitudes. Because holes retained from the previous ramp partially screen the surface, the initial electric field in Si is reduced at the start of the subsequent cycle. This screening slows the apparent pre-integration response: as shown in the inset, during successive pulsed-illumination events the charge transient requires an additional ≈1 µs to relax back to the dark level relative to the preceding cycle, even though individual carriers traverse the surface potential well in only tens of picoseconds to a few nanoseconds. The microsecond-scale recovery is instrument-limited rather than transport-limited, arising from measurement parasitics—principally the RC loading of pads and cabling and the finite bandwidth of the transimpedance-amplifier chain—which low-pass filter the measured current. The progressive attenuation in $I_{Dis}$ amplitude mirrors the cumulative decrease in $I_{Gr}$, evidencing trap filling and partial charge retention that lower the screening efficiency over successive sweeps.

The $I-V_{Ramp}$ representation highlights that the system’s photo-capacitive behavior is non-volatile on the timescale of the ramp cycle yet incremental across cycles, resembling analog memory characteristics. The observed hysteresis and amplitude roll-off therefore signify a deep-depletion photogating regime governed by trap-mediated charge integration. The correlation between the decreasing $I_{Dis}$ peaks and the systematic down-shift of $I_{Gr}$ baselines provides quantitative evidence for interfacial trap occupation and photo-induced threshold-voltage drift in the underlying MOS capacitor.

Such behavior is characteristic of photo-capacitive neuromorphic elements, where the charge integration and retention dynamics emulate synaptic weight modulation. The coupling of vertical charge storage to lateral conductance tuning thus establishes a functional pathway toward graphene-coupled optoelectronic synapses and integrating phototransistors.

**Figure 4:**
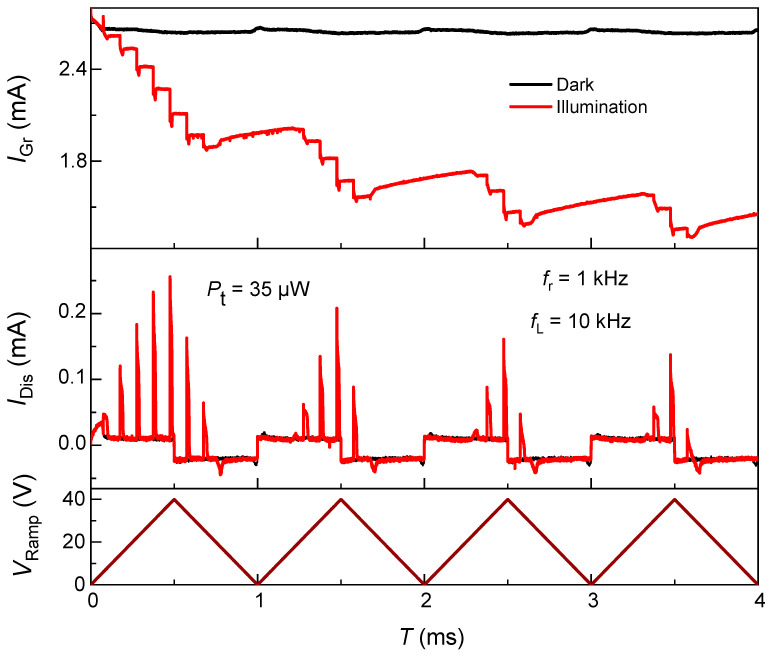
Pulse-resolved photogating and light-induced flat-band voltage shift in a graphene/SiO_2_/n-Si heterostructure. Top: Graphene channel current ($I_{Gr}$) at $V_{DS}=1\;V$ under dark (black) and pulsed 915 nm illumination (red). Middle: Corresponding displacement current ($I_{Dis}$) recorded through the Si/SiO_2_ stack. Bottom: Applied ramp voltage ($V_{Ramp}$, 0–40 V, $f_{r}=1\;kHz$) and laser modulation ($f_{L}=10\;kHz$). Multiple laser pulses within each ramp cycle produce discrete step-wise reductions in $I_{Gr}$ and synchronized $I_{Dis}$ spikes, evidencing incremental accumulation of photogenerated holes at the Si/SiO_2_ interface.

Figure 4 presents the time-resolved response of the graphene/SiO_2_/n-Si heterostructure when the pulsed 915 nm illumination is modulated at ten times the ramp-voltage frequency ($f_{L}=10\;kHz$). The experimental biasing remains identical to the earlier measurements: the silicon substrate is driven by a 0–40 V triangular ramp at $f_{r}=1\;kHz$ while the graphene channel is biased laterally at $V_{DS}=1\;V$. This configuration allows the system to experience multiple light-pulse excitations within a single cycle, thereby enabling direct observation of cumulative photogating and dynamic flat-band voltage shifts.

In the dark, $I_{Gr}$, remains nearly constant at ≈ 2.7 mA throughout the ramp, confirming the absence of field-induced modulation. Under illumination (red), $I_{Gr}$ exhibits a staircase-like decrease during each ramp period. Each discrete step coincides with a laser pulse, indicating that individual optical bursts incrementally increase the interfacial hole charge at the Si/SiO_2_ boundary. Because the vertical electric field persists over several light pulses before reversing, these photogenerated holes accumulate sequentially, progressively shifting the effective $V_{FB}$ of the MOS structure toward more positive bias. This shift manifests as the gradual suppression of graphene conductance within a single ramp and its incomplete recovery during the subsequent cycle. The observed step-wise current reduction thus represents real-time evidence of photo-induced $V_{FB}$ drift, directly supporting the interfacial photogating model established earlier.

The middle panel displays the corresponding $I_{Dis}$, which shows sharp transient peaks synchronized with the individual light pulses. The amplitude of these peaks diminishes gradually within each ramp period, reflecting the partial screening of the vertical field as interfacial charge builds up. The periodic recurrence of these spikes across the 10 kHz illumination sequence demonstrates that the oxide–interface system behaves as a photo-capacitive integrator: each pulse injects a quantized packet of charge, and the total integrated charge defines the instantaneous potential landscape experienced by the graphene channel. Importantly, as the ramp voltage resets, a portion of this charge remains trapped, producing a measurable offset in $I_{Dis}$ at the start of the next cycle—a signature of persistent flat-band voltage shift and trap retention.

The photo-inactive window—defined as the ramp gating interval during multi-pulse laser illumination in which no $I_{Dis}$ spikes appear and $I_{Gr}$ remains essentially flat—widens with successive ramps. For the first three cycles (↓/↑ denote falling/rising $V_{Ramp}$ segments), the window boundaries are (↓17 V, 0.77 ms)–(↑17 V, 1.22 ms), (↓24 V, 1.70 ms)–(↑23.5 V, 2.29 ms), and (↓27 V, 2.66 ms)–(↑27.8 V, 3.45 ms). This right-shift occurs because retained photocharge $\left(+Q_{it}\right)$ screens the surface field, requiring a larger gate bias to re-establish depletion/deep-depletion (the potential well separating carriers) and enable photo-injection. Once the charging window opens, the peak $I_{Dis}$ associated with the illumination pulse at 39.5 V diminishes across cycles—approximately 255 µA → 208 µA → 161 µA → 137 µA for cycles 1→4—because the retained charge from the previous ramp cycle by offering field screening reduces the vertical field and narrows the deep-depletion width, compromising hole integration. The graphene response mirrors this attenuation: for the same pulse count and optical power, the stepwise decrements in $I_{Gr}$ decrease from ~0.25 mA in cycle 1 to ~0.20, ~0.17, and ~0.16 mA at comparable points in later cycles, and the early-ramp flat segments indicate bias ranges with negligible photo-ionization in Si. Consequently, under identical illumination, both $I_{Dis}$ and $I_{Gr}$ exhibit delayed onset and reduced amplitude as cycles progress—direct evidence of field shielding by trapped holes at the Si/SiO_2_ interface.

These results provide direct, time-resolved proof that the light-induced modulation of the graphene conductance originates from minority-carrier integration in deep depletion, rather than from transient hot-carrier or bolometric effects in graphene. The correlation between the pulse-synchronized $I_{Dis}$ spikes and the step-wise decrements in $I_{Gr}$ establishes a self-consistent electrostatic coupling mechanism, wherein each photogenerated hole packet incrementally alters the Si/SiO_2_ interfacial potential. Consequently, the observed periodic yet cumulative evolution of $I_{Gr}$ represents an optical analogue of synaptic potentiation, with the shift in $V_{FB}$ serving as the internal memory variable.

### 3.4. Reset-Free Operation: Device B

**Figure 5:**
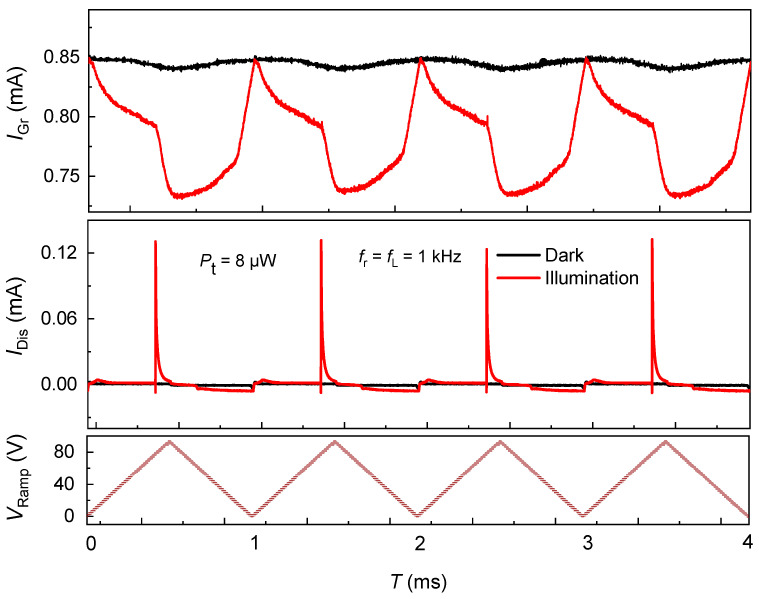
Instantaneous photogating without retention in a graphene/SiO_2_/n-Si device. Top: $I_{Gr}$ at $V_{DS}=1\;V$ under dark (black) and 915 nm illumination at 8 μW (red). Middle: $I_{Dis}$ through the Si/SiO_2_ stack. Bottom: applied $V_{Ramp}$ (0–93 V, ≈ 1 kHz; slope ≈ 186 kV/s). Illumination induces a reversible, in-ramp suppression of graphene conductance via photogating in deep depletion, but $I_{Gr}$ returns to the same baseline each cycle and $I_{Dis}$ spikes are unchanged, evidencing minimal trap-assisted retention and no measurable inter-cycle flat-band shift—consistent with a low $D_{it}$/fast-kinetics interface.

Figure 5 presents the transient response of a second graphene/SiO_2_/n-Si device measured with an expanded ramp range (0–93 V at 1 kHz) under 915 nm illumination while the graphene channel is held at $V_{DS}=1\;V$. In contrast to the first device, the dark $I_{Gr}$ trace is flat and the illuminated $I_{Gr}$ recovers to the same baseline at the start of each ramp, and the cycle-indexed curves nearly overlap. The $I_{Dis}$ shows reproducible charging/discharging transients whose peak amplitudes change little from cycle to cycle. Together these features indicate that, although photogenerated holes still integrate at the Si/SiO_2_ interface during deep depletion and capacitively gate the graphene within a ramp, little charge is retained once the field resets. The system therefore exhibits instantaneous photogating with negligible memory on millisecond timescales.

Electrostatically, this behavior corresponds to minimal light-induced flat-band shift between ramps: ${\Delta V}_{FB}^{\left(n+1\right)}-{\Delta V}_{FB}^{\left(n\right)}\approx 0$. Physically, it is consistent with a lower interfacial trap density ($D_{it}$) and/or faster detrapping/recombination at the Si/SiO_2_ boundary—fast compared to the 1 ms ramp period—so that the photogenerated interfacial charge annihilates during the down-sweep or immediately after ramp reversal. The near-constancy of $I_{Dis}$ peaks across cycles corroborates this interpretation: since $I_{Dis}=C_{eff}\left(V\right)\;dV_{Ramp}/dt$, comparable peak areas imply comparable charge transfer per cycle, i.e., no cumulative trap filling. A back-of-the-envelope estimate using $C_{eff}\approx {\;I}_{Dis}^{Peak}/\left(dV_{Ramp}/dt\right)$ with ${\;I}_{Dis}^{Peak}\approx 120–140\;\mu A$ and dV/dt= 1.86 × 10^5^ V/s yields $C_{eff}\approx$ 0.6–0.8 nF (device-level), consistent with a clean oxide stack; importantly, this capacitance does not evolve with cycle index, in contrast to the memory-bearing device. Notably, despite the larger maximum field (∼0.93 MV/cm across 100 nm SiO_2_), the absence of hysteresis suggests improved oxide/interface quality (fewer slow states), effective surface passivation, or shorter minority-carrier lifetime in Si that limits long-lived interfacial hole sheets.

Functionally, Device B therefore operates as a high-fidelity, reset-free photogated transistor: the in-ramp conductance reduction reflects the instantaneous interfacial charge, while the full recovery ensures repeatable cycles without drift. This contrasts with Device A’s trap-assisted retention (photo-capacitive memory). The pair thus bracket two desirable regimes—memory-bearing vs. memory-free photogating—controlled primarily by interface quality and trap kinetics.

## 4. Future Work and Outlook

The demonstrated platform supports two complementary application regimes. In the memory-free (reset-to-baseline) mode, the device functions as a fast, linear integrating photodetector: photogenerated charge is accumulated within each ramp and then fully erased, enabling accurate pulse counting, optical dosimetry, and high-dynamic-range exposure measurement in the NIR (850–1064 nm). In pixel form, $I_{Gr}$ serves as an exposure integrator while $I_{Dis}$ provides a built-in “exposure meter” for auto-gain control, well-suited to HDR imaging sensors and low-power optical front-ends. Because $I_{Dis}$ supplies precise timing markers and the graphene channel reports the stored charge state, the same pixel can support low-rate optical links (envelope/AM detection) and depth/spectral sensing by selecting the wavelength and ramp slope.

In the memory-bearing (trap-assisted retention) mode, the interface acts as an analog, leaky integrator, enabling optoelectronic synapses and in-sensor temporal filtering. Per-pulse $\Delta V_{FB}$ maps naturally to synaptic weight updates (short-/long-term plasticity), while brief accumulation or short-wavelength “scrub” pulses implement controlled erase/reset. This allows for programmable analog memory pixels that store scene history, compress exposure, or emphasize events/motion, as well as security-oriented PUFs and persistent dosimeters where partial retention encodes dose until reset. Practically, both regimes are CMOS-compatible (graphene on Si/SiO_2_), scalable to arrays, and tunable via interface passivation, oxide thickness/field, ramp waveform, and illumination spectrum/power. Moreover, graphene is now a technologically mature platform, with numerous reports demonstrating commercialization-oriented scalability, CMOS/BEOL-compatible processing, circuit-level integration, and long-term stability/passivation [43,44,45,46,47,48,49]. By design, this manuscript is a concept-level study: we focus on the device physics of dual-readout photogating and the distinction between memory-free and memory-bearing regimes, rather than evaluating scalability, system integration, or lifetime.

## 5. Conclusions

We investigated deep-depletion photogating in graphene/SiO_2_/n-Si using a dual-readout that simultaneously records the lateral $I_{Gr}$ and the vertical $I_{Dis}$. The method directly links photo-induced interfacial charge at Si/SiO_2_ to graphene conductance via ${\Delta V}_{FB}=Q/C_{ox}$. For a representative “Device A” operated with one optical pulse per ramp (0–40 V), the per-pulse flat-band shift decreases across four successive cycles from 38 → 31 V, consistent with progressive trap filling. The drop in injected charge between cycles yields retained charge of 0.18, 0.18, and 0.21 nC (cycles 1→ 2, 2→ 3, 3→ 4), equivalent to 2.1, 2 V, and 2.4 V of retained ${\Delta V}_{FB}$ and retention fractions of 5.6%, 5.7%, and 7.2%. The corresponding occupied interface-trap densities are $4.6\times 10^{11}\;{cm}^{-2},\;4.4\times 10^{11}\;{cm}^{-2},$ and $5.2\times 10^{11}\;{cm}^{-2}$, with a cumulative $1.4\times 10^{12}\;{cm}^{-2}$ over the four plotted cycles; the overall memory index is 17.4%. A second device (“Device B”) exhibits reset-to-baseline behavior with negligible inter-cycle drift, indicating a cleaner/faster interface. This time/voltage-resolved, two-current approach provides quantitative, CMOS-compatible metrology for tuning between instantaneous photogating and analog, trap-assisted memory in graphene–silicon platforms.

## Data Availability

The measured and analyzed data presented in the study is available upon reasonable request.
